# Minimizing Ochratoxin A Contamination through the Use of Actinobacteria and Their Active Molecules

**DOI:** 10.3390/toxins12050296

**Published:** 2020-05-05

**Authors:** Ixchel Campos-Avelar, Alexandre Colas de la Noue, Noel Durand, Blandine Fay, Véronique Martinez, Angélique Fontana, Caroline Strub, Sabine Schorr-Galindo

**Affiliations:** 1 Qualisud, Univ Montpellier, CIRAD, Montpellier SupAgro, Univ d’Avignon, Univ de La Réunion, F-34398 Montpellier, France; noel.durand@cirad.fr (N.D.); blandine.fay@umontpellier.fr (B.F.); veronique.martinez@umontpellier.fr (V.M.); angelique.fontana@umontpellier.fr (A.F.); caroline.strub@umontpellier.fr (C.S.); sabine.galindo@umontpellier.fr (S.S.-G.); 2 CIRAD, UMR Qualisud, F-34398 Montpellier, France

**Keywords:** *Streptomyces*, fungi, mycotoxins, enzymes, biodegradation, detoxification

## Abstract

Ochratoxin A (OTA) is a secondary metabolite produced by fungal pathogens such as *Penicillium* *verrucosum*, which develops in food commodities during storage such as cereals, grapes, and coffee. It represents public health concerns due to its genotoxicity, carcinogenicity, and teratogenicity. The objective of this study was to evaluate the ability of actinobacteria and their metabolites to degrade OTA and/or to decrease its production. Sixty strains of actinobacteria were tested for their ability to prevent OTA formation by in vitro dual culture assays or with cell free extracts (CFEs). In dual culture, 17 strains strongly inhibited fungal growth, although it was generally associated with an increase in OTA specific production. Seventeen strains inhibited OTA specific production up to 4% of the control. Eleven actinobacteria CFEs reduced OTA specific production up to 62% of the control, while no substantial growth inhibition was observed except for two strains up to 72% of the control. Thirty-three strains were able to degrade OTA almost completely in liquid medium whereas only five were able to decrease it on solid medium, and two of them reduced OTA to an undetectable amount. Our results suggest that OTA decrease could be related to different strategies of degradation/metabolization by actinobacteria, through enzyme activities and secretion of secondary metabolites interfering with the OTA biosynthetic pathway. CFEs appeared to be ineffective at degrading OTA, raising interesting questions about the detoxification mechanisms. Common degradation by-products (e.g., OTα or L-β-phenylalanine) were searched by HPLC-MS/MS, however, none of them were found, which implies a different mechanism of detoxification and/or a subsequent degradation into unknown products.

## 1. Introduction

As climate change progresses, the presence of fungi increases and with them, the occurrence of mycotoxins, which are estimated to contaminate about 25% of global food crops [1]. These secondary metabolites raise huge concerns due to their harmful effects on human and animal health [2] and to their relative stability to industrial processing [3,4,5]. *Penicillium verrucosum* is a mycotoxinogenic fungus that develops mostly on stored seeds and is able to produce ochratoxin A (OTA) [5,6]. OTA has carcinogenic, hepatoxic, immunosuppressive, and genotoxic effects; in addition to potential nephrotoxic and teratogenic incidences [7,8], food authorities have established regulatory measures to limit the use and consumption of contaminated raw materials [9,10].

Facing the millions of tons of grains lost per year due to the presence of mycotoxins, several mitigation techniques are currently under evaluation. At this time, prevailing physical processes include manual sorting, sieving, density segregation, washing, dehulling, steeping, milling, extruding, and heating [11]. Another physical decontamination approach is the use of binding matrices that are able to adsorb toxic molecules such as activated charcoal, bentonite, hydrated aluminum silicates, and zeolites [12]. Emerging techniques such as pulsed light, cold plasma, and gamma irradiation have proven to be efficient to reduce ochratoxin A in nuts and seeds, but the absence of toxic by-products remain to be evaluated before applying these practices during food processing [13,14]. Chemical options consist of acidic and alkaline treatments (e.g., ammoniation) as well as oxidizing (e.g., ozonation) and reducing agents, but these treatments are currently not authorized in the European Union for the decontamination of human food commodities [15].

Aside from physical and chemical treatments, biological solutions include the use of microorganisms and their enzymes [16]. Several microorganisms are able to inhibit mycotoxin biosynthesis and to detoxify mycotoxins once they are produced, either by binding them to their cell wall or by degrading them into less toxic compounds, or a combination of several mechanisms [17,18,19]. The primary approach is the direct application of detoxifying microorganisms to the contaminated food matrix, but the biological agent must already be recognized as safe for consumption (GRAS) (e.g., lactic bacteria and yeasts) [20]. For microorganisms able to detoxify mycotoxins but not GRAS, their direct utilization in the food matrix is not allowed. Thus, the search for enzymes implied in their degrading capacities can lead to interesting applicative alternatives [21]. Some enzymes known to be involved in ochratoxin A degradation are carboxypeptidases, deoxygenases, lipases, amidases, and proteases [22].

Actinobacteria are filamentous, Gram-positive bacteria that are found in terrestrial and aquatic ecosystems. They are mainly of interest in biotechnology and agriculture due to the huge diversity of enzymes, antibiotics, and antifungal compounds they can produce [23,24]. Along with their antifungal properties, actinobacteria have been repeatedly shown to be able to degrade OTA [16,17] and they can produce enzymes capable of degrading aromatic compounds [25,26]. The main OTA biodegradation mechanism consists of the hydrolysis of the amide bond via hydrolytic enzymes (e.g., carboxypeptidase A), which results in its two primary degradation by-products: OTα and L-β-phenylalanine, which are reportedly much less toxic than OTA [15,16]. Another, more hypothetical, mechanism is the cleavage of the lactone ring, giving place to an OTA with an open lactone, however, this process is reversible [27]. To confirm the effectiveness of the detoxification, the identification of the degradation by-products is crucial. HPLC MS and HPLC MS/MS are well suited for the research, identification, and quantification of degradation by-products [28].

Aside from potential degradation mechanisms, some authors have shown that some strains belonging to the *Streptomyces* genus possess the ability to bind mycotoxins onto their cell walls [17]. In addition, *Streptomyces* strains were found to reduce the expression of the *acpks*, *acOTApks*, *acOTAnrps*, and *veA* genes, which modulate the biosynthesis of OTA in *Aspergillus carbonarius* [17]. These remarkable abilities make actinobacteria promising tools for biodetoxification, and although their direct application in food matrices is not authorized, they can be the source of various enzymes and metabolites of interest for the reduction of mycotoxins’ associated risk.

The aim of our work was to study the interactions between 60 *Streptomyces* strains isolated from compost and amended soil with *P. verrucosum* and its toxin. In a first step, *Streptomyces* strains were confronted with *P. verrucosum* to evaluate their inhibitory effect on growth and OTA specific production (OTAsp). In a second step, we designed experiments to gain further information on the specific interactions occurring during dual culture assays. The CFEs were added to the growth media of *P. verrucosum* to understand the role of constitutive *Streptomyces* metabolites on inhibiting or promoting fungal growth and/or OTA biosynthetic pathway. Interactions with OTA were studied by adding the toxin to the media during *Streptomyces* growth, and also in their CFEs to understand if OTA reductions could originate from its degradation by secreted enzymes or through its metabolization. All our data were then processed using clustering analysis (heatmap) and Pearson’s correlation with the aim of segregating strains with comparable profiles of interest. Finally, OTA degradation was investigated by HPLC-MS/MS in order to draw assumptions about potential degradation by-products typically described in the literature.

## 2. Results

### 2.1. Antagonistic Evaluation of Actinobacteria and Their Cell Free Extracts (CFE)

**Figure 1:**
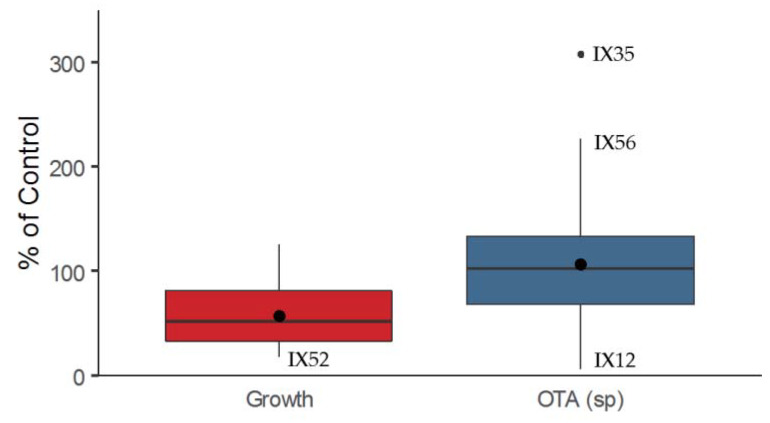
Effect of the 60 strains of actinobacteria in dual culture assay with *Penicillium verrucosum* on fungal growth and ochratoxin A (OTA) specific production. Boxplot represents the distribution of the data expressed as % of a control (fungal growth and specific mycotoxin production without actinobacteria).

Sixty strains of actinobacteria were tested by in vitro dual culture assays against *P. verrucosum* on CYA (Czapek Yeast extract Agar) medium at 25 °C for 12 days. Growth was reduced down to 20% of the control with a mean of 58% (median 53%). Ochratoxin A (OTA) specific production decreased to 7% of the control. Nevertheless, 27 strains increased OTA specific production, especially IX56 (227%) and IX35 (307%) (Figure 1). Of note, *Streptomyces* strains with no impact on pathogen growth were more efficient at decreasing OTA specific production, whereas those with strong growth inhibition properties often enhanced mycotoxin production. Strains IX02, IX09, and IX52 were the only strains able to inhibit both *P. verrucosum* growth (38%, 35%, and 19% of the control, respectively) and OTA specific production (51%, 29%, and 46% of the control, respectively).

**Figure 2:**
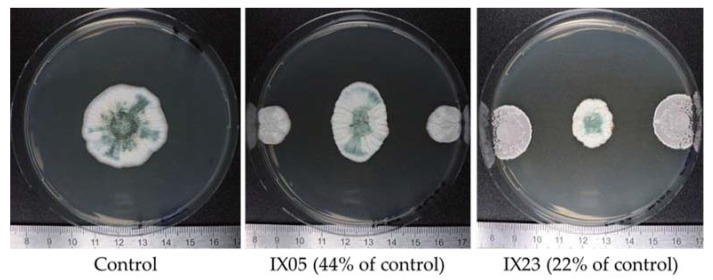
Example of inhibition profiles observed during dual culture assays of actinobacteria strains against *P. verrucosum* on CYA plates at 25 °C for 12 days.

During the dual culture assays, different inhibition profiles were observed. As shown in Figure 2, the *P. verrucosum* colony had either an oval or round shape, probably reflecting the diffusion and efficacy of antifungal metabolites in the agar.

**Figure 3:**
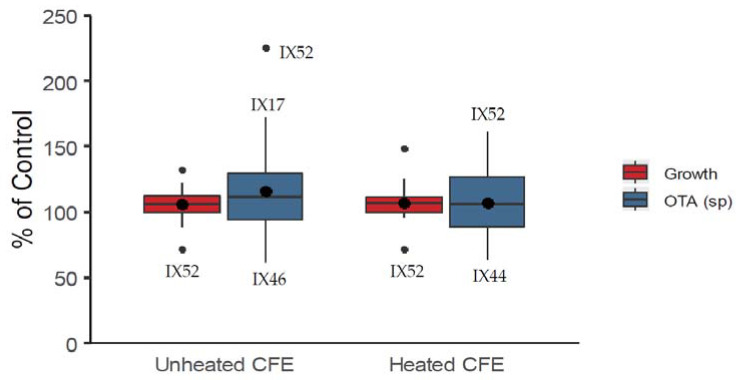
Effect of the 60 cell free extracts (CFEs) added at 10% in the culture medium of *Penicillium verrucosum* on growth and OTA specific production. Boxplot represent the distribution of the data expressed as % of a control (fungal growth and specific mycotoxin production without CFEs). Thermal treatment for heated CFEs: 100 °C-10 min.

CFEs were produced with CYB (Czapek Yeast extract Broth) liquid culture of actinobacteria grown for five days at 25 °C under agitation (180 rpm) and added at a concentration of 10% in the solid growth medium of *P. verrucosum*. CFEs were much less efficient at reducing both growth and OTA specific production of *P. verrucosum* than that observed in dual culture (Figure 3). Only one CFE (IX52) inhibited *P. verrucosum* growth substantially down to 72% of the control, regardless of whether it underwent a thermal treatment or not (mean = 106%, median = 106% for unheated CFEs and mean = 107%, median = 107% for heated CFEs). OTA specific production was reduced by a few strains up to 62% (IX46) and 64% (IX44) of the control by unheated and heated CFEs, respectively (mean = 116%, median = 112% for unheated CFEs and mean = 107%, median = 106% for heated CFEs). However, half of the CFEs increased OTA specific production during fungal growth. As observed earlier, the most effective CFEs for the reduction of fungal growth were also the ones promoting OTA specific production. For example, CFEs of strain IX52 decreased *P. verrucosum* growth to 72% of the control (with and without thermal treatment), while simultaneously causing an overproduction of OTA with 225% and 161% of the control, for unheated and heated CFEs, respectively.

### 2.2. Mycotoxin Degradation Assay

**Figure 4:**
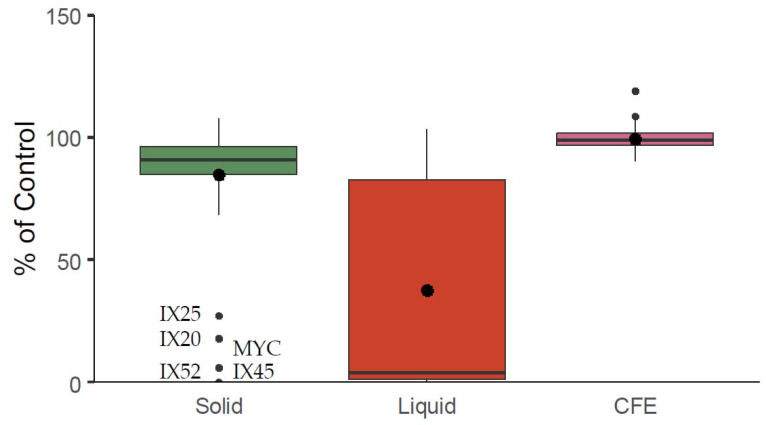
Degradation of ochratoxin A by bacterial cells after 10 days of culture at 25 °C in solid and liquid medium, and by unheated CFEs after 48 h at 25 °C under agitation (180 rpm). Boxplots represent the distribution of the degradation efficiency given in % of the control. The control was performed by adding the toxin in CYA or CYB medium without bacteria and incubated in the same conditions as the samples.

The ability of the sixty strains of actinobacteria to degrade mycotoxin was evaluated by culturing them in solid CYA and liquid CYB medium supplemented with OTA. A concentration of ~280 ng/mL was used because it is representative of the maximal toxin concentration typically encountered for *P. verrucosum* alone after 12 days on CYA plates at 25 °C. Furthermore, CFEs were tested for degradation capacities by spiking OTA in CFEs. The control was performed by adding the toxins in CYA medium without bacteria and incubated in the same conditions as the samples. The results are presented in Figure 4. OTA was strongly degraded by only five strains on solid medium (IX25 < IX20 < MYC < IX52 < IX45) from 27% to 0% of the control (mean = 85%, median = 91%), while 33 strains degraded it almost entirely in liquid medium (mean 38%, median 4%). Negligible OTA degradation was noted with a maximum of 90% of the control (mean = 99%, median = 99%) for the unheated CFEs. Thus, heated CFEs were not tested for the degradation assay.

### 2.3. Global Analysis of Strains: Heatmap Analysis and Pearson Correlation Index

**Figure 5:**
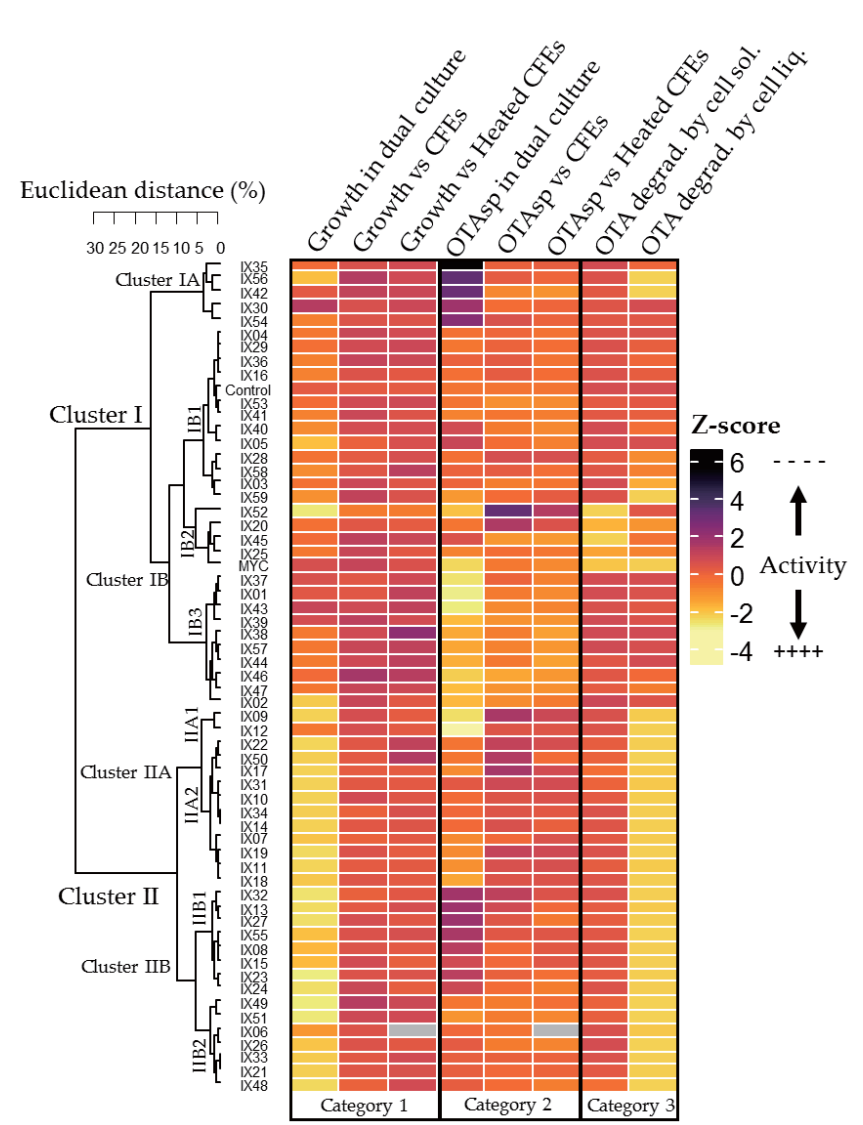
Summary heatmap of the effect of actinobacteria and their CFEs on *Penicillium verrucosum* growth (Category 1) and OTA specific production (Category 2) along with the bacteria’s ability to degrade OTA in solid and liquid medium (Category 3). The results are given in a range of colors according to their Z-score where purple to black indicates a weak activity or an increase compared to the control and yellow indicates the strongest activity. Missing values are indicated in gray.

**Figure 6:**
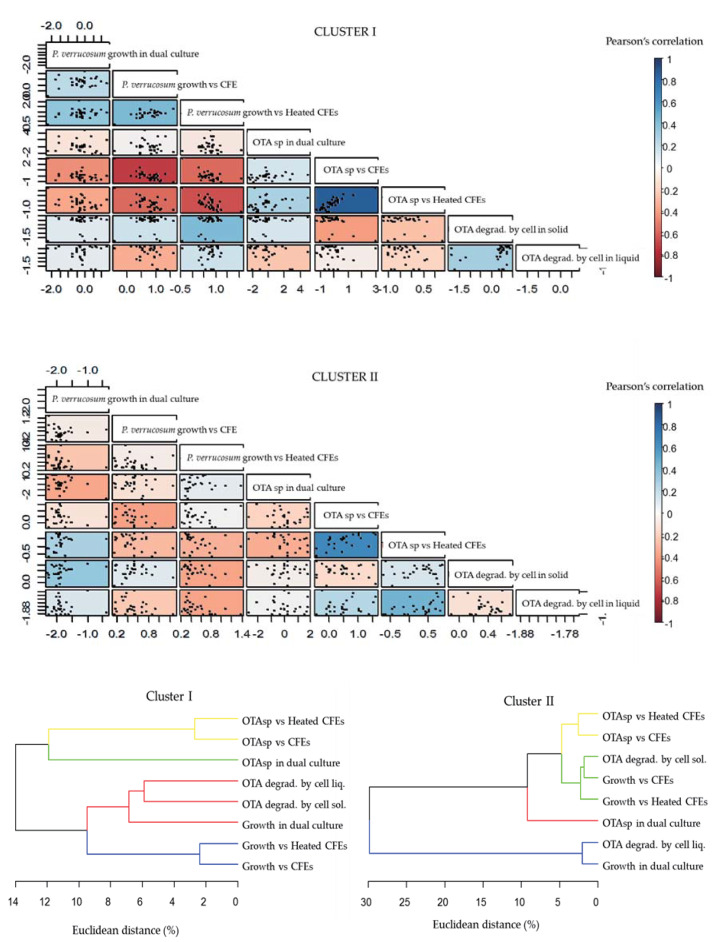
Pearson’s correlation of the effects of actinobacteria and their CFEs in *Penicillium verrucosum* growth and OTA specific production along with the bacteria’s ability to degrade OTA in solid and liquid medium. In the correlation scale, the 1 in blue indicates a strong positive correlation and the −1 in dark red is a strong negative correlation. The correlation was calculated for the strains of each main cluster of the heatmap (Cluster I and Cluster II). Dendrograms in the lower part show the relationship and proximity in % of Euclidean distance of each of the studied parameters for Clusters I and II. Different groups are indicated by different colors.

To gain better insight into the specific features of the 60 strains and their potential correlations, all our results were analyzed using a clustering analysis with a heatmap representation (Figure 5) and a correlational analysis through the calculation of the Pearson’s correlation index for the two main clusters (Figure 6).

The heatmap depicts the effect of each bacteria regarding three different types of interactions:

- *P. verrucosum* growth in dual culture or with CFE added to the medium (Category 1);
- OTA specific production during *P. verrucosum* growth confronted with bacteria, or with CFE added to the medium (Category 2); and
- OTA degradation by actinobacteria in solid and liquid medium (Category 3).

OTA degradation by CFEs was not included in the analysis because it was found earlier to be ineffective for all our strains. In order to compare different parameters with variable distributions, a standardization was made by converting the original values in % for each test to Z-scores, calculated independently within each of the three previously established categories. The stronger the activity regarding fungal growth, inhibition of OTAsp, or OTA degradation capability, the lower the values of Z-score. Finally, the Z-score was color coded accordingly: clear yellow represents a strong activity (low Z-score) while dark purple to black represents an enhancement of fungal growth or OTAsp compared to the control (high Z-score). Intermediate colors between these extremes represent moderate or no activity. Finally, Euclidean distances were calculated considering the strains’ whole profile within each category and sub-category, and further transformed as the percentage of similarity before being clustered accordingly. The main features of each group were identified by plotting boxplots of the different categories for each subcluster (Supplementary Materials Figure S1). Strains were grouped in two main clusters, I and II. Cluster I is characterized by strains with a mitigated effect on *P. verrucosum* growth and contrasting impacts on OTA specific production, either by cells or by CFEs. Strains from Cluster IA provoked a strong increase in OTA specific production while Cluster IB was subdivided in three distinct clusters. Cluster IB1 had no or moderate enhancing effect on OTA specific production, while subcluster IB3 showed a mild to strong ability to decrease it. Five strains (IX20, IX25, IX45, IX52, and MYC) grouped in subcluster IB2 were the only ones able to degrade OTA significantly on a solid medium even if their degradation efficiency was reduced in the liquid medium. Cluster II contained strains with high inhibition rates of *P. verrucosum* and strong capacities to degrade OTA in liquid medium. Furthermore, most of the strains from Cluster IIB caused a moderate (IIB2) to strong (IIB1) increase in OTA specific production, whereas the subcluster IIA showed mild (IIA2) to strong (IIA1) inhibition of the latter. For the sake of clarity, a summary of the main features for each cluster depicted in the heatmap is presented in Table 1.

We performed a Pearson correlation test between all the experimental parameters studied, which is illustrated in Figure 6. All correlation values and their significance are presented in the Supplementary Materials (Table S1). The Pearson correlation coefficient of Figure 6 illustrates a strong positive correlation between the effect of heated and unheated CFEs on OTA specific production for both clusters (0.9 for Cluster I, *p* < 0.001and 0.7 for Cluster II, *p* < 0.001). However, their low efficiency could explain the strong correlation observed. These results confirm that the degradation through enzymatic activity might not play a significant role in the apparent reduction of OTA specific production by CFEs. Moreover, the absence of the degradation of OTA in CFEs confirms this assumption. There was a strong negative correlation of the effect of CFEs under both treatments on pathogen growth with its OTA specific production in Cluster I (between −0.6, and −0.7, all with *p* < 0.001), which indicates that when the CFEs of this cluster were efficient at inhibiting fungal growth, they usually provoked an increase in OTA specific production. In Cluster II, only CFE activity on growth and OTA production were significantly correlated with a negative correlation of −0.4 (*p* = 0.04). Moderate positive correlations were identified between the OTA specific production with heated CFEs and the degradation of OTA by cells in liquid medium in Cluster II (0.5, *p* = 0.01). A low but significant positive correlation was observed in Cluster I between the growth of *P. verrucosum* in dual culture and the growth with heated CFEs (0.4, *p* = 0.04) as well as the effect between heated and non-heated CFEs on pathogen growth (0.4, *p* = 0.01).

The Pearson correlation coefficient between the growth of *P. verrucosum* and its OTA specific production was almost null for Cluster I (−0.1). For Cluster II, moderate negative correlation −0.4 (*p* = 0.054) between the growth inhibition rate and the increase in OTA specific production was noted, showing that stressing conditions might lead to an enhanced OTA production. In fact, a few strains in Cluster II reduced OTA specific production while they inhibited *P. verrucosum* growth (e.g., IX09 and IX51). No significant correlation was observed in Cluster I where subcluster IA (Figure 5) strongly enhanced OTA specific production, even if their effect on fungal growth remained limited.

The dendrograms at the lower part of Figure 6 show how the effect of actinobacteria on the experimental parameters were related within each cluster. Parameters were more closely related in Cluster I than in Cluster II, with a maximum Euclidean distance of 14% and 30%, respectively, which could explain why the correlations were stronger in Cluster I. We can also observe that the parameter association varied slightly between the two clusters. In Cluster I, the specific production of OTA in dual culture was quite distant (12%) from the specific production of OTA with the CFEs, while in Cluster II, they were separated for only 5%. Additionally, degradation of OTA in both mediums were quite close in Cluster I (6%), but the distance increased to 30% in Cluster II. Pathogenic growth either in dual culture or with heated and unheated CFEs was separated by 9% in Cluster I while in Cluster II, there seemed to be a substantial difference between the growth of the pathogen in dual culture and that with CFEs (30%). The relationship between the OTA specific production with heated and unheated CFEs was the only one that remained constant, with about 3% of separation in both clusters.

**Figure 7:**
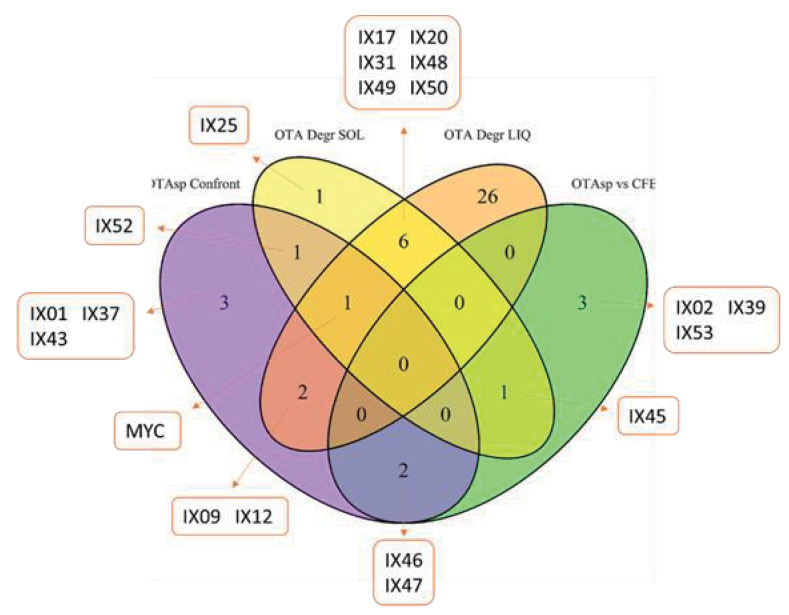
Venn diagram of the effect of actinobacteria and their CFEs in the specific production of OTA during *in vitro* dual culture assays and their ability to degrade pure OTA in solid and liquid medium.

The Venn diagram of Figure 7 was constructed with strains able to strongly decrease OTA, either by reducing its specific production or by degrading it (at least 50% for dual culture assays and liquid degradation and at least 20% for unheated CFEs and solid degradation). Seven strains were able to degrade OTA both in solid and liquid medium: IX17, IX20, IX31, IX48, IX49, IX50, and Mycostop^®^. From these, Mycostop^®^ was also able to decrease OTA specific production during dual culture assays. We also noted that the two strains were able to decrease OTA specific production in both dual culture assays and by their CFEs: IX46 and IX47. Two strains that decreased OTA specific production were also able to degrade the mycotoxin in liquid medium: IX09 and IX12. Only IX45 could degrade OTA in a solid medium while being able to decrease OTA specific production with its CFEs.

### 2.4. Search for Degradation by-Products by HPLC

During the last phase of our work, we focused on the search of the main by-products of OTA biodegradation. Three strains with strong degradation abilities in liquid medium were selected from Cluster II (i.e., IX23, IX31, and IX48). They were cultured for three and seven days at 25 °C in CYB liquid medium with ~280 ng/mL of OTA. Main degradation by-products resulting for OTA degradation by microorganisms and already identified in the literature were searched using HPLC MS/MS: OTα and L-β-phenylalanine generated by the hydrolysis of the amide bond of OTA [15,25,29], and lactone opened form of OTA (OP-OTA) produced though the hydrolysis of the lactone ring of OTA [28,30,31]. While a strong degradation of OTA at three and seven days was observed as found earlier for these three strains, none of the common by-products were identified (OTα, L-β-phenylalanine, OP-OTA). Thus, we also proceeded to research the precursors of the OTA biosynthetic pathway such as OTB, which results from the dechlorination of the isocoumarin moiety [16], OTβ, mellein, viomellein, penicilic acid, and asperlactone [32,33]. However, none of them was identified.

## 3. Discussion

During this work, we evaluated the ability of sixty strains of actinobacteria to inhibit the growth of the mycotoxinogenic fungus *Penicillium verrucosum* and to minimize the occurrence of ochratoxin A, either by preventing its production or by degrading it. *Penicillium verrucosum* appears mostly in temperate and colder zones and is one of the main contaminants of stored grains in Europe [34,35], which makes it a main concern for detoxification research. Studies on the antagonistic effects of *Streptomyces* have been mostly conducted on *Aspergillus* sp. [17,36]. Even if the OTA biosynthetic pathway gene cluster is close between *Aspergillus* sp. and *Penicillium* sp., as already suggested in the literature [32,37], an accurate comparison of the regulation of their biosynthetic pathway under similar conditions is scarce. For example, *Penicillium verrucosum* has been shown to switch between OTA and citrinin production under oxidative stress or light [35,38,39] or in response to high osmolarity to adapt to various environments, whereas important *Aspergillus* producers such as *A. carbonarius* do not show such features [40]. Whether *Aspergillus* sp and *Penicillium* sp. OTA producing species behave similarly when exposed to biological stress remains to be elucidated. The wide picture of actinobacteria vs. *Penicillium* sp. interactions presented in this study might represent a good opportunity to select strains with varying effects on *P. verrucosum* growth and OTA production, in order to compare the behavior of *Aspergillus* sp and *Penicillium* sp under similar biological stressing conditions.

Results of the dual culture assays seem to indicate that in some cases, *Streptomyces* metabolites with an effect on *P. verrucosum* growth also led to an enhanced OTA specific production. Correlation analysis confirmed the negative relationship between these two observations for Cluster II with the most representative group being Cluster IIB1. However, the most important increases observed for OTA specific production were related to strains with no effect on growth (Cluster IA). A similar effect was observed for CFEs, regardless of being heated or not, which induced OTA specific production while no substantial effect on growth was observed, even if the thermal treatment somewhat lowered the observed effects. Thus, it seems likely that their effects on OTA specific production were not due to complex molecules such as heat sensitive enzymes, but rather to thermostable antifungal metabolites.

An explanation could be that OTA was overproduced by *P. verrucosum* when the fungus encountered stress conditions such as the presence of inhibitory compounds as a defense mechanism to secure its environmental niche, as suggested earlier by Venkatesh and Keller [41]. It has been demonstrated that several fungi such as *Aspergillus flavus* and *Aspergillus carbonarius* typically overproduce aflatoxins and OTA, respectively, when exposed to low concentrations of fungicides [42]. Thus, special attention must be paid to select strains that do not enhance mycotoxin production when an effect on fungal growth is desired, such as strains from Cluster IIA and especially IIA1 (IX09), which decrease both growth and OTA production. However, the use of specific compounds inhibiting fungal growth raises concern about the potential colonization of the ecological niche by other harmful microorganisms that could also represent a threat for food safety [36]. For future research, strains from Cluster IB3 that have no impact on *P. verrucosum* growth, but are able to strongly reduce OTA specific production, represent very interesting candidates. The inhibition of OTA specific production with no effect on growth might be explained by an interference between secondary metabolites and OTA biosynthetic pathway, as reported by EL Khoury et al., who showed that *Streptomyces* strains were able to diminish the expression of regulation genes of OTA biosynthesis in *Aspergillus carbonarius*. However, this effect was not attributed to a specific molecule [17,36]. Other similar results were found with other microorganisms such as yeast, which decrease the expression of the *pks* gene and subsequent OTA production [43]. For aflatoxin, identified molecules such as blasticidin and aflastatin A have been described as repressors of the aflatoxin gene cluster, leading to a subexpression of the enzymes responsible for aflatoxin production, without necessarily affecting fungal growth [44]. A similar mechanism acting on the regulation of OTA biosynthetic pathway for *P. verrucosum* seems likely and remains to be verified. Additionally, active secondary metabolites secreted by actinobacteria that have an effect on the OTA biosynthetic pathway could be identified in the future. This complementary research will allow for a refinement of our understanding of mycotoxin biosynthesis inhibition when no effect on growth is observed.

CFEs were less efficient than the bacteria themselves to inhibit *P. verrucosum* growth. In a similar trend, CFEs decreased OTA specific production to a lower degree. However, only one concentration of 10% of CFEs in the media was used during the screening and further studies are required to evaluate the potential of CFEs at higher concentrations. Another assumption could be that secondary metabolites are produced in higher quantity when the bacteria is in contact with the fungi. Many recent studies have highlighted that the co-culture of microorganisms can lead to an overproduction of secondary metabolites [45,46,47,48]. Thus, the secretion of OTA or other secondary metabolites by the fungi could enhance the secretion of antifungal compounds or antitoxinogenic compounds by actinobacteria.

Regarding the reduction of OTA during degradation assays, we excluded a binding effect considering that sonication, cellular disruption, and vortexing were performed during mycotoxin extraction. In addition, our extraction solvent was methanol, which is also used in protocols of pellet washing to recover OTA potentially bound by actinobacteria [17,25], making toxin adsorption mechanisms very unlikely in our conditions. A large disparity between the degrading efficacy on solid and liquid media was observed, as OTA was much more effectively degraded in liquid medium. Such differences were also observed by Varga et al. [49] when testing OTA biodegradation by *Aspergillus* species. Better access to the substrate (OTA) in liquid medium could be a simple explanation as the mycotoxin is constantly in contact with the bacteria during agitation. Interestingly, most strains of Cluster II with strong growth inhibition properties were also able to degrade OTA in liquid medium, but none of them could degrade it on solid medium. Indeed, our results raise some interesting questions about the very few strains from Cluster IB2 that were able to degrade OTA with higher efficiency on solid media. Reports of the enhanced production of enzymes on solid media have been reported in the literature [50] and could explain why some strains exhibited higher degradation rates in such conditions. However, this observation was uncommon for the 60 strains of actinobacteria in this study (33 strains with strong degrading ability in liquid medium vs. five strains in solid medium).

The low OTA reduction efficiency in CFEs compared to the degradation by actinobacteria was in accordance with the results of El Khoury et al. [17] and suggests that degradation may be achieved by intracellular enzymes. Such intracellular enzymes with strong OTA degrading ability into OTα have been described for *Alcaligenes faecalis*, a Gram-negative bacteria isolated from soil [51], but not for actinobacteria, as far as we know. Some actinobacteria of other genera such as *Brevibacterium* are able to degrade OTA into OTα and L-β-phenylalanine, probably through the action of a carboxypeptidase [25]. Further studies are required in order to identify the active enzymes of the strains that strongly degraded OTA and their intra or extracellular location, since it is well known that actinobacteria are able to produce proteases and peptidases that can be used for biodegradation purposes [25]. Another assumption about the CFEs’ inefficiency regarding OTA degradation could be that their production is promoted by the presence of the toxin. To our knowledge, the impact of OTA on *Streptomyces* metabolism and exocellular enzyme production has so far not been studied. However, it has been demonstrated that chitinase production by *Streptomyces coelicolor* A3(2) is strongly enhanced in soil medium rather than in laboratory media, probably because of the presence of chitin [52]. As actinobacteria and many fungi share the same ecological niches, it would be interesting to study if mycotoxins could act as signaling molecules on *Streptomyces* metabolism, and if they could induce the production of enzymes responsible for their degradation.

No common degradation by-products (i.e., OTα, OTβ, L-β-phenylalanine) were identified by HPLC-MS/MS for the selected strains with strong degrading ability (i.e., IX23, IX31, and IX48), which suggests either a subsequent degradation into unknown products or a different mechanism of detoxification. From one microorganism to another, the OTA’s degradation mechanisms differed, as did the degradation by-products. This was confirmed by various studies where the degradation products could not be identified [16,18,30,53]. Shi et al. did not find any common degradation products when evaluating the degradation ability of OTA by Bacillus subtilis CW14 [18]. When studying the degradation of OTA by fungi, Bejaoui et al. observed the progressive reduction of OTα and the appearance of an unknown compound after nine days of culture [53] as did Varga et al. after seven days of culture [49]. Thus, in our case, the absence of commonly found by-products could also be due to the length of the incubation period (three and seven days), that possibly allowed a further decomposition into unknown molecules. Further studies could be performed with isotopically labeled OTA in order to elucidate the fate of the degraded mycotoxin [54]. Additionally, residual toxicity assays could be performed on HepG2 cells [55], or by in vivo systems like nematodes or zebrafish [56,57] to ensure the detoxification capacity of the studied actinobacteria strains.

## 4. Conclusions

In conclusion, several actinobacteria strains were able to minimize the occurrence of OTA either by preventing its formation, degrading it, or both mechanisms. The methodology employed in this study (screening by nine modalities, clustering and correlation analysis) helped easily distinguish the features of each strain and to group them into clusters of comparable abilities. A heatmap representation helped easily select specific strains according to the desired effect. For instance, we could choose strains that strongly inhibit OTA specific production without affecting *P. verrucosum* growth to preserve the ecological niche, or strains that decreased OTA specific production but had low degradation capacities, if we wish to deepen the study of the inhibition of the mycotoxin biosynthetic pathway. Remarkable observations such as the degradation ability of OTA on the solid medium were very scarce among the strains studied, found for only five strains among the sixty. Interesting correlations such as the increase in OTA specific production when growth was strongly reduced have also been observed. Thus, our collection represents a great start for further studies in order to identify active metabolites and enzymes, and to provide a better understanding of the various interactions that can exist between actinobacteria, *P. verrucosum*, and OTA. Among the three strains studied by HPLC MS/MS for OTA degradation, no common by-products were found.

## 5. Materials and Methods

### 5.1. Actinobacteria Strains

Fifty-nine actinobacteria strains were isolated from organic amendments and soil samples collected at the Hérault Department in the South of France. The strains constitute a collection maintained in the laboratory of the UMR QualiSud at the University of Montpellier. Mycostop^®^ (MYC) strain *Steptomyces griseoviridis* K61 was included in the tests as a commercialized biocontrol agent for comparison. A 16S preliminary identification demonstrated that all the strains in the collection belonged at 97% to the genre *Streptomyces* (data not shown). The strains were grown on ISP4 medium (10 g/L starch, 1 g/L K_2_HPO_4_, 1 g/L MgSO_4_, 2 g/L (NH_4_)_2_SO_4_, 1 g/L CaCO_3_, 1 mg/L FeSO_4_, 1 mg/L MgCl_2_, 1 mg/L ZnSO_4_, 18 g/L bacteriological agar, pH 7.2) for 11 days at 28 °C. Spores were then collected by scraping the surface of the Petri dish with 5 mL of distilled water +0.01% Tween 20 and filtered through sterile cotton. The spore suspensions were aliquoted, then stored at −80 °C.

### 5.2. Actinobacteria Spore Numeration by Flux Cytometer

For actinobacteria spore count, a Novocyte ACEA flux cytometer was employed. After counting, the results were validated by colony forming units on CYA medium after 11 days of culture. Before each test, bacterial spore suspensions were unfrozen and diluted to a concentration of 10^7^ spores/mL.

### 5.3. Pathogen Strains

A mycotoxinogenic strain of *Penicillium verrucosum* NRRL5571, kindly provided by Dr. Olivier Puel from INRAE’s UMR Toxalim [54], was employed to carry out this work. Fungus was maintained on PDA (Biokar BK095HA) inclined tubes covered in paraffin oil prior to use. For fungal spore harvesting, pathogens were grown on PDA plates for seven days at 25 °C, then spores were scraped from the surface by adding 10 mL of distilled sterile water and filtered through sterile cotton. Pathogen spores were harvested and enumerated before each test.

### 5.4. Antagonistic Evaluation

#### 5.4.1. Actinobacteria Cells

Antagonistic assays were performed on Czapek Yeast Extract Agar medium (CYA: 30 g/L sucrose, 5 g/L yeast extract, 1 g/L K_2_HPO_4_, 0.3 g/L NaNO_3_, 0.05 g/L KCl, 0.05 g/L MgSO_4_, 1 mg/L FeSO_4_, 1 mg/L ZnSO_4_, 0.5 mg/L CuSO_4_, 15 g/L agar, pH ~7.4), which allowed both pathogen and bacteria proper growth and sporulation without favoring one over the other.

Dual culture assays were implemented on Petri dishes by inoculating 10 µL of the actinobacteria spore suspension at 10^7^ CFU/mL on each side of the plate. After three days at 25 °C, 10 µL of the pathogen spore suspension at 10^6^ CFU/mL were inoculated at the center of the plate and left for nine more days at 25 °C before image analysis and mycotoxin extraction. As a control, the pathogen was inoculated on a CYA plate without actinobacteria. All tests were done in triplicate.

#### 5.4.2. Cell Free Extracts

Actinobacteria strains were cultured on Czapek Yeast Extract Broth (CYB: 30 g/L sucrose, 5 g/L yeast extract, 1 g/L K_2_HPO_4_, 0.3 g/L NaNO_3_, 0.05 g/L KCl, 0.05 g/L MgSO_4_, 1 mg/L FeSO_4_, 1 mg/L ZnSO_4_, 0.5 mg/L CuSO_4_, pH ~7.4) medium for five days at 25 °C and 180 rpm. Then, liquid cultures were centrifuged at 10,000× *g* for 5 min and the supernatant was filtered through a PES (Polyethersulfone) filter at 0.22 µm to eliminate bacterial cells. The resulting cell free extracts (CFEs) were used to evaluate the effect of bacterial metabolites on the growth and mycotoxin production of the fungal pathogen by adding 10% of each CFE in the CYA medium for *P. verrucosum*, then 10 µL of the pathogen spore suspension at 10^6^ CFU/mL was inoculated at the center of the plate and left for 12 days at 25 °C. In order to identify a potential effect of actinobacteria extracellular enzymes, CFEs were also tested after a heat treatment at 100 °C for 10 min. As a control, the pathogen was inoculated on a CYA plate without CFEs. All tests were done in triplicate.

#### 5.4.3. Pathogen Surface Growth Measurement and OTA Specific Production Calculation

Surface growth of *P. verrucosum* colonies was measured in ImageJ software (1.52a, Wayne Rasband National Institute of Health, Bethesda, MD, USA, 2018). Growth inhibition was established in comparison to the control without actinobacteria, which represented 100% of growth. Results are given in % of control.

(1) $$Growth\;ratio\;\left(\%\;of\;control\right)=\frac{Assay\;area\;\left(cm^{2}\right)}{Control\;area\;(cm^{2})\;}\times 100$$

OTA specific production was calculated by dividing the mycotoxin amount in the whole sample by the area of the fungal colony.

(2) $$OTA\;specific\;production\;\left(OTAsp\right)=\frac{Amount\;of\;mycotoxins\;produced\;\left(ng\right)}{Colony\;area\;\left(cm^{2}\right)}$$

Then, the percentage of reduction for OTA specific production was calculated in comparison to the control without actinobacteria.

(3) $$OTA_{sp}\;\left(\%\;of\;control\right)=\frac{OTA_{sp}\;assay}{OTA_{sp}\;control}\times 100$$

### 5.5. Mycotoxin Degradation Assay

#### 5.5.1. Screening of OTA Degradation by Actinobacteria and Their CFEs

Degradation assays were performed using ochratoxin A (Sigma Aldrich, St. Quentin Fallavier, France) suspended in acetonitrile in order to prepare a stock solution of 31 µg/mL. OTA concentration in the control was about ~280 ng/mL. Mycotoxin was added to liquid CYB and solid CYA medium, followed by the addition of 20 µL of actinobacteria spore suspension at 10^7^ spores/mL and incubated at 25 °C with an agitation at 180 rpm for the liquid cultures. After 10 days, mycotoxin was extracted and analyzed as described before. CFEs, produced as described before, were also tested for their ability to degrade OTA after being in contact with the mycotoxin for 48 h at 25 °C and 180 rpm. As a control, mycotoxin was spiked in solid CYA or liquid CYB medium and incubated following the same incubation protocols as the samples.

(4) $$OTA\;degradation\;\left(\%\;of\;control\right)=\frac{OTA_{ng/mL}\;assay}{OTA_{ng/mL}\;control}\times 100$$

#### 5.5.2. Degradation Assays for the Search of Degradation by-Products

A selection of three strains with strong degradation abilities in liquid medium was made (IX23, IX31, and IX48). These were cultured in liquid medium with OTA (~280 ng/mL) as described in Section 5.5. for three and seven days at 25 °C and 180 rpm. Controls included liquid CYB medium alone and bacteria cultured in liquid CYB without OTA, both incubated under the same conditions to eliminate potential unknown molecules corresponding to bacterial metabolites and/or culture medium. Main degradation by-products of OTA, namely OTα, L-β-phenylalanine, and lactone opened OTA (OP-OTA) as well as biosynthesis precursors such as OTB, OTβ, mellein, viomellein, penicilic acid, and asperlactone were further searched using HPLC-MS/MS.

### 5.6. Mycotoxin and Degradation By-Products Analysis

#### 5.6.1. Sample Extraction

For the mycotoxin extraction of the antagonistic and CFE assays, the whole content of the Petri dish, liquid culture or CFE was transferred into a plastic container. Agar medium was cut into small pieces with a scalpel before extraction. After weighing the sample, acidified methanol (3.85% formic acid) was added to the sample with a ratio of 1.5/5, followed by 20 min of agitation at 250 rpm. A total of 500 µL of the mixture were evaporated in a Speed Vac (Eppendorf^®^ AG, Hamburg, Germany) at 60 °C until dryness, and then 2 mL of the mobile phase (methanol/water/acetic acid, 69:30:1) were added. To ensure that mycotoxin was not bonded to the cells, samples underwent 20 min of sonication, followed by 10 min in a cell disruptor, and finally thoroughly mixed in a vortex before filtering through a 0.45 µM PTFE (Polytetrafluoroethylene) filter for the HPLC analysis.

For HPLC MS/MS, sample extraction was performed similarly, except for the last step of resuspension after evaporation, where the added mobile phase was ultra-pure water acidified with acetic acid (0.5%).

The percentages of recovery for OTA were 97% and 95% after the extraction and resuspension step for fluorescence HPLC and HPLC MS/MS, respectively. The by-product recovery rate was not established because the analysis was only qualitative.

#### 5.6.2. OTA Quantification Using Fluorescence HPLC

OTA concentration for all experiments was quantified by HPLC using a fluorescence detector (Shimadzu RF 20A, Japan). The operating conditions were as follows: injection volume of 100 µL; C18 reverse-phase HPLC column, Uptisphere type, ODS, 5 µm particle size, 5 ODB, 250 × 4.6 mm, with identical pre-column, thermostatically controlled at 35 °C; isocratic flow rate of 1 mL/min (mobile phase: methanol/water/acetic acid, 69:30:1); excitation wavelength of 333 nm and emission wavelength of 460 nm. The concentrations were calculated from a calibration curve established from an OTA standard (1 µg/mL; ref PD 226 R. Biopharm Rhône Ltd., Glasgow, UK). Detection and quantification limits were established at 0.085 and 0.25 ng/mL, respectively.

#### 5.6.3. Search for Degradation By-Products by HPLC-MS/MS

A qualitative analysis was performed on HPLC MS/MS for the research of degradation by-products. The OTα standard was purchased from Romerlabs (Getzersdorf, Austria) and OTB from Sigma Aldrich (St. Quentin Fallavier, France). OTβ was synthetized from OTB as described by Harris and Mantle [58]. Briefly, 800 µL of OTB standard solution (5µg/mL) was hydrolyzed by reflux with 50 mL of HCl 6 M for 18 h and extracted with ethyl acetate. Mellein was extracted from a seven-day old plate of *Aspergillus ochraceus*, as described by Zhang et al. [28].

The MS/MS parameters (MRM, EC) for metabolites investigated were taken from the following articles:

- OTA, OTα, OTB, and mellein “Zhang et al.” [28]
- OTβ “Wang et al.” [32]
- L-β-phenylalanine “Patharajan et al.” [30]
- Lactone opened OTA “Bazin et al.” [31]
- Viomellein [33]
- Penicilic acid [33]
- Asperlactone [33]

Detection of mycotoxin and their by-products were achieved using Ultra High Performance Liquid Chromatography (UHPLC, Shimadzu, Tokyo, Japan) coupled with a mass spectrometer (8040, Shimadzu, Tokyo, Japan). LC separation was performed using a Phenomenex Kinetex XB Column C18 (50 mm × 2 mm; 2.6 µm particles) at 50 °C with an injection volume of 50 µL. Mobile phase composition was (A) 0.5% acetic acid in ultra-pure water and (B) 0.5% acetic acid in isopropanol (HPLC MS grade, Sigma, St Louis, MO, USA). A mobile phase gradient program was started at 90% A (0.01 min), 45% A at 1.5 min, 15% A at 3.5 min, 20% A at 4 min, 98% A at 4.01 min, and finally 98% A at 11 min, and the mobile phase flow rate was 0.4 mL min ^−1^. The mass spectrometer was operated in electrospray positive (ESI+) and negative (ESI–) ionization mode, and two multiple reaction monitoring (MRM) transitions for each analyte were monitored for quantification and qualification. OTA detection and quantification limits were established at 0.06 and 0.2 ng/mL, respectively. As only qualitative measurements were performed for the degradation by-products, their limits of detection and quantification were not established. All data were analyzed using LabSolution Software (v5.91/2017, Shimadzu, Tokyo, Japan,2017).

### 5.7. Data Analysis and Data Visualization

Preprocessing of the data for the heatmap consisted of a conversion of percentages to Z-scores calculated as follows: Z−score=(X−μ)/σ, where X is the measurement, μ is the mean value of the effect of all strains in a category (fungal growth, OTA specific production, OTA degradation), and σ is the standard deviation of the value of the effect of all strains in the considered category. This conversion allowed all data results within each category to be normalized in order to compare them, as the results of the different categories evaluated had variable distributions. Therefore, with the standardized data, the default Euclidean method was chosen for measuring the distance between the clusters, which is expressed as a percentage of similarity of the effect of actinobacteria regarding the different parameters. Boxplots and Pearson correlation graphics were developed in Rstudio.

## Figures and Tables

**Table 1:** Summary of specific features observed during the clustering analysis of the impact of the screened actinobacteria and their cell free extracts (CFEs) on *P. verrucosum* growth and ochratoxin A (OTA) specific production.

Cluster	Specific features	Subcluster		Specific Features
I	Limited effect on *P. verrucosum* growth (dual culture and CFEs)	A	-	Highest increase in OTA specific production (dual culture) Two strains degrading OTA in liquid medium (IX56, IX42)
		B	1	No or moderate increase in OTA specific production
			2	Only cluster with strains able to highly degrade OTA on solid medium Strong degradation capacities in liquid medium
			3	High inhibition of OTA specific production (dual culture and CFEs)
II	Strong effect on *P. verrucosum* growth (dual culture) High degradation of OTA in liquid medium	A	1	Highest inhibition of OTA specific production (dual culture)
			2	Moderate inhibition of OTA specific production (dual culture)
		B	1	Strong increase in OTA specific production
			2	Moderate increase of OTA specific production (dual culture and CFEs)

## Supplementary Materials

The following are available online at https://www.mdpi.com/2072-6651/12/5/296/s1, Figure S1: Boxplot of the effect of actinobacteria and CFEs on *P. verrucosum* growth and OTA specific production. Each boxplot represents a subcluster of the heatmap from Figure 5. GDC = *P. verrucosum* growth in dual culture, GCFE = *P. verrucosum* growth vs. cell free extracts (CFEs), GHCFE = *P. verrucosum* growth vs. Heated CFEs, OTADC = OTA specific production (sp) in dual culture, OTACFE = OTAsp vs. CFEs, OTAHCFE = OTAsp vs. Heated CFEs, OTADS = OTA degradation by cells in solid medium, OTADL = OTA degradation by cells in liquid medium, Table S1: Values of the Pearson correlation between the measured parameters during the screening of the effect of actinobacteria and their CFEs on *P. verrucosum* growth and OTA production.
