# Supplementary material for: Minimizing Ochratoxin A Contamination through the Use of Actinobacteria and Their Active Molecules
Source: Toxins (Basel). 2020 May 5;12(5):296. doi: 10.3390/toxins12050296 (PMC7290465; doi:10.3390/toxins12050296)
Supplement: Supplementary file 1 [file toxins-12-00296-s001.pdf]

Supplementary Materials: Minimizing Ochratoxin A Contamination through the Use of Actinobacteria and Their Active Molecules

Ixchel Campos-Avelar, Alexandre Colas de la Noue, Noel Durand, Blandine Fay, Véronique Martinez, Angélique Fontana, Caroline Strub and Sabine Schorr-Galindo

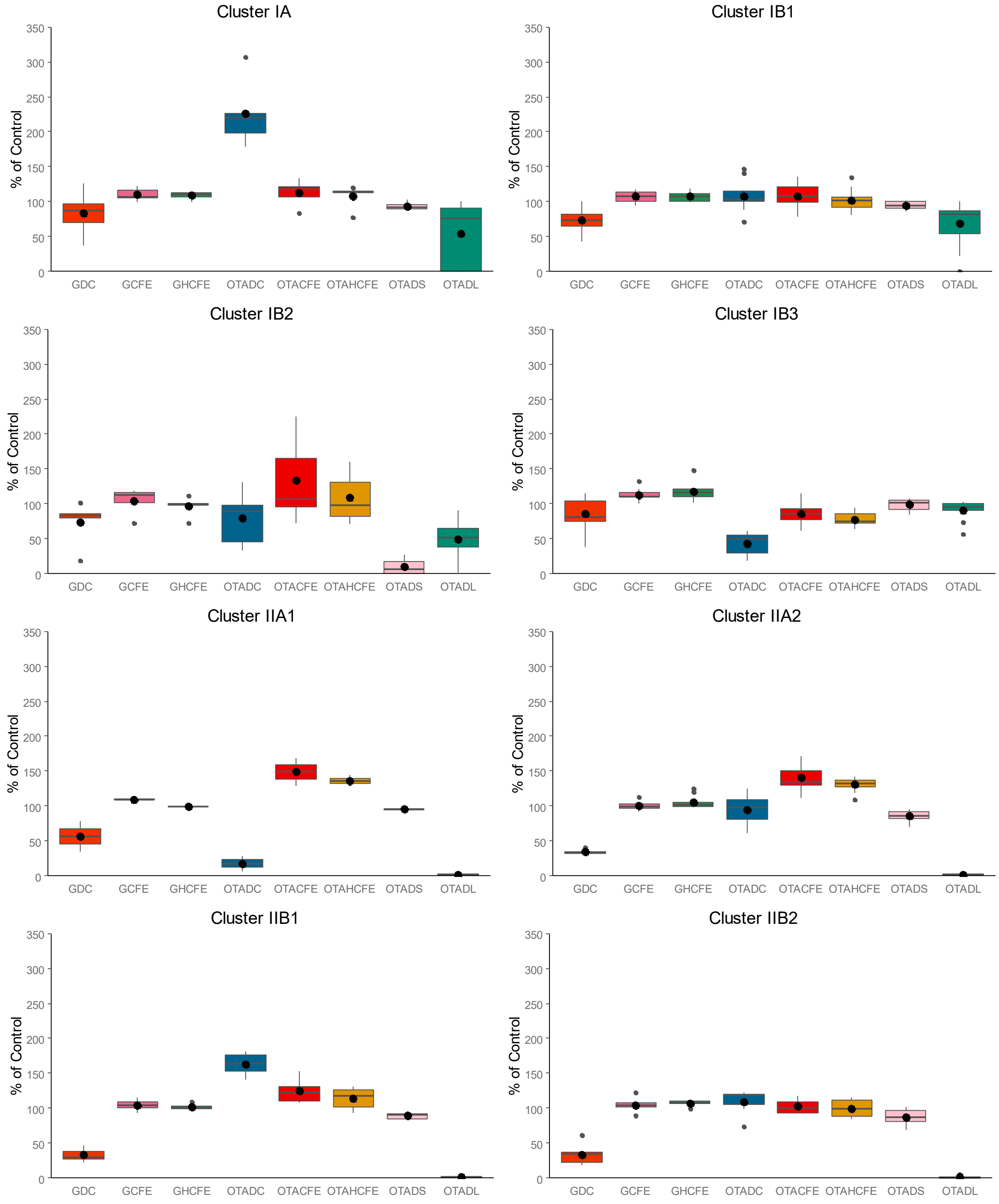

Figure S1. Boxplot of the effect of actinobacteria and CFEs on *P. verrucosum* growth and OTA specific production. Each boxplot represents a subcluster of the heatmap from Figure 5. GDC = *P. verrucosum* growth in dual culture, GCFE = *P. verrucosum* growth vs Cell Free Extracts (CFEs), GHCFE = *P.*

verrucosum growth vs Heated CFEs, OTADC = OTA specific production (sp) in dual culture, OTACFE = OTAsp vs CFEs, OTAHCFE = OTAsp vs Heated CFEs, OTADS = OTA degradation by cells in solid medium, OTADL = OTA degradation by cells in liquid medium.

Table 1. Values of the Pearson correlation between the measured parameters during the screening of the effect of actinobacteria and their CFEs on *P. verrucosum* growth and OTA production.

	GDC	GCFE	GHCFE	OTADC	OTACFE	OTAHCFE	OTADS	OTADL	
Cluster I	1								GDC
	0.2 (0.18)	1							GCFE
	0.4 (0.04)*	0.4 (0.01)*	1						GHCFE
	−0.1 (0.59)	0 (0.1)	−0.1 (0.73)	1					OTADC
	−0.4 (0.01)*	−0.7 (<0.001)***	−0.6 (<0.001)***	0.1 (0.47)	1				OTACFE
	−0.4 (0.04)*	−0.6 (<0.001)***	−0.7 (<0.001)***	0.3 (0.11)	0.9 (<0.001)***	1			OTAHCFE
	0.1 (0.65)	0.2 (0.37)	0.4 (0.01)*	0.1 (0.49)	−0.4 (0.02)*	−0.3 (0.14)	1		OTADS
	0.1 (0.72)	−0.3 (0.053)	0.2 (0.37)	-0.2 (0.17)	0 (0.86)	−0.2 (0.30)	0.3 (0.08)	1	OTADL
Cluster II	1								GDC
	0 (0.80)	1							GCFE
	−0.2 (0.22)	0 (0.86)	1						GHCFE
	−0.4 (0.054)	−0.1 (0.60)	0.1 (0.74)	1					OTADC
	−0.1 (0.64)	−0.4 (0.04)*	0 (0.10)	−0.2 (0.32)	1				OTACFE
	0.3 (0.15)	−0.3 (0.14)	−0.3 (0.10)	−0.3 (0.08)	0.7 (<0.001)***	1			OTAHCFE
	0.4 (0.06)	0.1 (0.68)	−0.4 (0.054)	0 (0.89)	−0.1 (0.47)	0.1 (0.54)	1		OTADS
	0.1 (0.55)	−0.2 (0.22)	−0.4 (0.052)	0 (0.95)	0.3 (0.18)	0.5 (0.01)*	−0.1 (0.60)	1	OTADL

GDC = *P. verrucosum* growth in dual culture, GCFE = *P. verrucosum* growth vs Cell Free Extracts (CFEs), GHCFE = *P. verrucosum* growth vs Heated CFEs, OTADC = OTA specific production (sp) in dual culture, OTACFE = OTAsp vs CFEs, OTAHCFE = OTAsp vs Heated CFEs, OTADS = OTA degradation by cells in solid medium, OTADL = OTA degradation by cells in liquid medium. *P*-values are indicated in the parenthesis. * *P<0.05*, *** *P<0.001.*
